# The microbiome and non-occupational human-animal interactions: a scoping review

**DOI:** 10.3389/fpubh.2026.1938888

**Published:** 2026-09-08

**Authors:** Emilie M. MacInnis, Tatum Ebbeskotte, Ahmed AbdelKhalek, Leanne O. Nieforth

**Affiliations:** 1 Department of Comparative Pathobiology, College of Veterinary Medicine, Purdue University, West Lafayette, IN, United States; 2 John Martinson Honors College, Purdue University, West Lafayette, IN, United States; 3 Department of Human Development and Family Science, College of Health and Human Sciences, Purdue University, West Lafayette, IN, United States

**Keywords:** companion animal, human animal interaction, human microbiome, microbiome composition, One Health

## Abstract

Human-animal interactions may impact the composition of the human microbiome. This can then influence human physical, mental, and social wellbeing. This scoping review synthesizes the literature on the impacts of intentional, non-occupational human-animal interactions on the human microbiome. Six databases (PubMed, CINAHL, PsycINFO, Web of Science, CAB Abstracts, and ProQuest Dissertations and Theses) were searched using keywords for human-animal interactions and microbiomes. Database searching resulted in 2,946 publications. Publications were screened according to the following inclusion criteria: (1) published in English, (2) peer-reviewed article or dissertation presenting empirical data, (3) studies humans engaging in intentional, non-occupational interaction with animals, and (4) compares human microbiome composition before and after human-animal interactions or compares humans that interact with animals to humans that do not interact with animals. Screening resulted in 30 relevant publications. Of these, 24 reported a significant effect of human-animal interactions on microbiome composition. However, there were notable gaps in the reporting of demographics and methodology, such as length of human-animal interaction, species of animal, and variable 16S regions of focus. To better understand the impacts of human-animal interactions on the human microbiome, future studies should include detailed reporting of both human-animal interactions and microbiome analysis methods.

## Introduction

There is a growing interest in the interconnectedness of people, animals, plants, and the environment, often referred to as One Health or Traditional Ecological Knowledge (1, 2). While this interconnectedness is often discussed in the context of zoonosis, the microbiome is beginning to be recognized as an important and potentially beneficial aspect of One Health (3). The human microbiome is the collective community of microorganisms inhabiting a specific human tissue (e.g., gut, skin, mucous membrane) (4). The composition of human microbiomes may influence the physical, mental, and social wellbeing of their host (4, 5). This composition can be impacted by factors external to the host, such as interactions with other people, animals, and the environment (1, 6, 7). Understanding the impacts of these interactions on the composition of human microbiomes may provide a deeper understanding of the interconnected mechanisms of human wellbeing.

Of particular interest are the impacts of interactions between humans and animals on the human microbiome. Humans seem innately drawn to animals and may intentionally engage in human-animal interactions (HAIs) (8). Interacting with a companion animal or pet is one of the most common forms of HAI, with an estimated 60% of households containing at least one companion animal (9). However, HAIs take many forms, including, but not limited to companion animals, animal-assisted therapy, animal-assisted education and humane education, employment that involves working with animals, and human-wildlife interactions (10). Given the scope of HAIs and the potential impacts of the microbiome on human health, it is important to understand the relationship between HAIs and the human microbiome.

### Microbiome and human health

Microbiomes have the potential to impact the health of their human host (4, 5). Numerous public health concerns including obesity, diabetes, cancer, food allergies, depression, and Alzheimer's Disease have been linked to microbiome composition, particularly the gut microbiome (4). Microbiome composition can be influenced by environmental factors such as animal exposure, antibiotic use, sexual activity, smoking, dietary choices, socioeconomic environment, and built environment (6, 11). Continuous particle shedding during human and animal activities leads to microbial exchange through vectors like dust, air, water, food, and physical touch (4, 12–14). Due to the vast number of environmental influences on the microbiome, it is important to understand how these changes impact human health.

Some environmental conditions that impact microbiome composition have been characterized in the current literature. Residents of urban environments display lower microbial biodiversity than those in rural environments (15). This difference can lead to immature immune systems and delayed organ development for urban residents (15). In rural environments, farmers have shown differences in microbiome composition based on which livestock they work with (12). Additionally, the length of interaction with livestock impacts the magnitude of the difference (12). Early life exposure to microbes on farms correlates to a decreased likelihood of developing diseases such as atopy and asthma (11).

As with farmers and livestock, humans can exchange microbiota with their companion animals, impacting both the human's and animal's health (1). Living with a dog or cat has been linked to greater biodiversity within the microbiome (1). However, the type and amount of exchanged microbes vary significantly depending on environmental factors and which microbiomes are studied (1). On the contrary, some studies have found mixed results or no significant changes due to interactions with companion animals, showcasing inconsistency in the current literature (16–18). With risks of confounding environmental variables, small sample sizes, and a lack of agreement on what comprises a “healthy microbiome,” it is difficult to understand the extent of the impact that interacting with animals has on the human microbiome (1). These conflicting studies highlight a need to synthesize the literature to identify avenues for further understanding the impacts that HAIs have on the human microbiome.

### Human-animal interactions

Interactions between people and animals range from encounters with wildlife to cohabitating with companion animals (10). However, recurring, long-term interactions with animals are of particular interest for understanding the impact of HAIs on the microbiome due to correlations between the length of HAIs and the impact on microbiome composition (1). These recurring interactions would include interactions such as with companion animals, service animals, or animals involved in animal-assisted education (e.g., classroom pets).

Companion animals are commonly referred to as pets and include dogs, cats, birds, fish, rabbits, reptiles, and small rodents (19). Service animals, sometimes known as assistance animals, are animals that perform specific tasks to help one person manage their disability (20). In the United States, service animals must be dogs or miniature horses (20). They often undergo extensive training to partner with a handler and perform needed support tasks for vision, mobility, or psychiatric support (10). Animal-assisted education involves incorporating animals into formal or informal educational programs (21). This can take many forms, such as incorporating pets into classrooms, participating in humane education courses at an animal shelter, or reading to a dog at a library (21).

Interacting with animals can improve human psychological, physiological, and social wellbeing (10). Socially, HAIs encourage human-to-human interaction and increase the feeling of being part of a community (10, 22). In particular, those with service dogs report being greeted and approached more in public than those without service dogs, combating feelings of loneliness and isolation (22, 23). Reported psychological benefits include alleviating loneliness, improving feelings of self-worth, and improving symptoms of psychiatric disorders (10, 24). Improvements in executive functioning, attention, and motivation may also be correlated with HAIs (10). Physiological health benefits include fewer doctor visits, higher survival rates from coronary artery disease, more frequent exercise, and decreased blood pressure (25, 26). Despite these benefits, HAIs may also negatively impact human wellbeing. Common negative impacts include allergies, zoonosis, bite risk, caregiver burden, housing challenges, and financial impacts (27, 28). Ultimately, research indicates that for many people, the benefits of HAIs outweigh the costs and can directly yield physical, social, and psychological benefits (25, 29).

In addition to these direct impacts, there is increasing evidence of the potential for HAIs to indirectly impact human wellbeing. Animals have the capacity to influence the environmental microbiome by introducing new beneficial bacteria to the environment, which can in turn influence the human microbiome (12, 13). Living with animals, such as on farms or with companion animals, leads to protective measures within the microbiome that may help reduce the risk of food allergies, atopy, asthma, and antibiotic resistance (30–32). These impacts on human health underscore the need to examine the current literature on HAIs' effects on the microbiome.

### Current review

Considering the impact that microbiome changes can have on human physical and mental health, it is crucial to understand how HAIs may impact the human microbiome. The primary focus of this scoping review was to summarize and synthesize literature focused on how human microbiomes may or may not change in the context of human-animal interactions. Given that humans and animals interact in a multitude of different ways on a daily basis, it was important to limit the scope of examined HAIs to comparable forms of interaction. This allows current evidence to be more accurately compared and synthesized. Consequently, the research question for this review was “What are the effects of intentional, non-occupational human-animal interactions on human microbiomes?” Intentional interactions include therapy animal visits, companion animal interactions, humane education, animal-assisted education, service dog partnerships, and volunteer work (21). Interactions due to the human's employment were excluded due to the potential for major differences between occupational and non-occupational interactions (e.g., condition of animal, length of interaction, type of animal, method of interaction, etc.). Narrowing the target of this scoping review to intentional, non-occupational HAI allowed for a more focused investigation of comparable literature.

A scoping review was chosen over a systematic review as scoping reviews are more appropriate for emerging topics, as they can more broadly identify the way research on the topic is being conducted (33). A formal, standardized assessment of quality, bias, or methodological limitations was not conducted, as these are not standard analyses for scoping reviews (33). In alignment with the purpose of scoping reviews, aims were developed that serve to clarify the scope of the current literature, identify gaps, and provide an overview of findings. The first aim of this review (Aim I) was to describe the composition of the literature related to intentional human-animal interaction and the microbiome. The second aim (Aim II) was to summarize the human-animal interactions being measured and the methods of measuring the microbiome. The third aim (Aim III) was to summarize the reported outcomes related to human-animal interaction and the microbiome.

## Methods

### Search strategy

**Figure 1:**
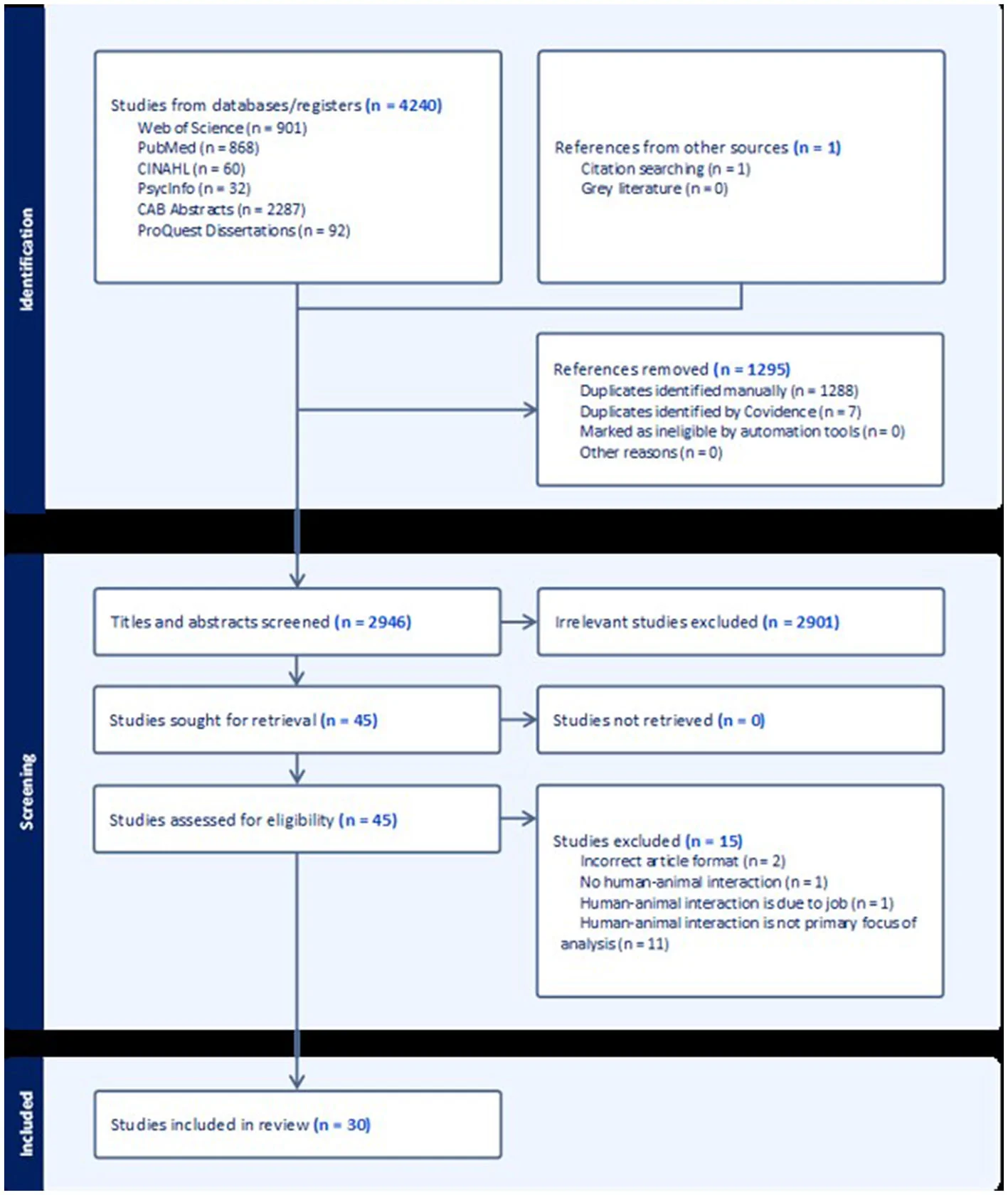
PRISMA flowchart summarizing the article collection and screening process for this scoping review.

A scoping literature review was conducted in accordance with the Preferred Reporting Items for Systematic Reviews and Meta-Analyses—Scoping Reviews (PRISMA-ScR) guidelines (34). The PRISMA flow chart in Figure 1 summarizes the article collection and screening process. Prior to conducting the literature search, all study procedures were established, including the search strategy, inclusion and exclusion criteria, and the data extraction procedure. This protocol was preregistered with OSF (Registration https://doi.org/10.17605/OSF.IO/CN6TM). A total of six databases were searched from the inception date to 21 February 2025 (PubMed, CINAHL, PsycINFO, Web of Science, CAB Abstracts, and ProQuest Dissertations and Theses). The search strategy was adapted to each database by mapping terms appropriately to each database's vocabulary, in consultation with an academic librarian. All database searches included identifiers for human-animal interactions (e.g., “companion animal”, “therapy horse”, “service dog”) and identifiers for human microbiomes (e.g., “microbiome”, “gut flora”, “microbiota”). The full list of identifiers is available in Supplementary materials. The database search resulted in 4,240 articles.

### Criteria and screening

After manual removal of duplicates in Zotero (*n* = 1,295), all remaining articles were uploaded to Covidence (35). Additional duplicate entries were automatically removed by Covidence (*n* = 11), resulting in a total of 2,946 articles that moved on to the screening process. See Table 1 for inclusion and exclusion criteria.

**Table 1:** Criteria for screening publications for inclusion or exclusion in the scoping review examining the impacts of human-animal interactions on the composition of the human microbiome, used during title/abstract screening and full text screening.

Criterion type	Inclusion criteria	Exclusion criteria
Language	Published in English	Published in any language besides English
Publication Type	Publication is a peer-reviewed journal article or dissertation/thesis presenting empirical data	Publication is not a peer-reviewed journal article or dissertation/thesis and does not present empirical data (e.g., theoretical articles, book chapters, reviews, conference presentations, commentaries)
Population	Humans interacting with animals	Animal-only studies
		Human-only studies
Interaction	Humans engaging in intentional, non-occupational interaction with animals	Humans interacting with animals due to their job
		Unintentional or accidental human-animal interactions (e.g. wildlife encounters)
		Studies with no interaction between humans and animals
Comparison	Human microbiome composition before and after interacting with animals OR humans that interact with animals compared to humans that do not interact with animals	Studies of human-animal interaction without consideration of human microbiomes
		Studies of only animal microbiomes

The research team, consisting of four people, first completed a round of title and abstract screening in which articles deemed irrelevant based on the title and abstract were removed. Once title and abstract screening concluded, the full texts of the remaining articles (*n* = 45) were reviewed for relevance, using the criteria outlined in Table 1, by two members of the research team. In each round of screening, two members of the research team reviewed each title and abstract or full text, respectively, and voted “Include”, “Exclude”, or “Maybe”. Conflicts occurred when the two people disagreed in their votes. Once a week the research team met for a conflict resolution meeting in which they discussed the conflicts and came to a conclusion as a team on how to vote. After screening was completed, the resulting articles (*n* = 29) were included in the scoping review. Additionally, backward and forward citation checking was conducted by one team member to capture any articles that were missed by the keyword search. This resulted in one additional article being included (36). While this article was published after the database search date, it was deemed appropriate to include because it was published only shortly after the database search and because inclusion would more accurately represent the breadth of literature on HAI and human microbiome composition.

### Data extraction

Following screening and citation checking, 30 articles remained that aligned with the inclusion criteria and explored the influence of human-animal interactions on human microbiomes. A pre-registered data extraction template was created prior to data extraction and loaded into Covidence, where it was used to extract data from each article. See Supplementary materials for the data extraction template. Two members of the research team collaborated to extract data. Data extracted for Aim I focused on the composition of the current literature and documented the research aim or hypothesis of the article, the title, name and country of the authors, year of publication, journal of publication, and demographic information on both human and animal participants. Data extracted for Aim II looked at the measures for HAI and the microbiome and included additional demographic information for human and animal participants, sample size, details on how samples were collected, handled, processed, and analyzed, study design and limitations, ethics approval, and information about the microbes present in both conditions. Data extracted for Aim III summarized the reported outcomes of the studies, including noting if there were differences in the microbiome between HAI and no HAI and the general outcomes. Once the data were extracted from all articles, one member of the research team reviewed and finalized the extracted data. Any discrepancies in extracted data were discussed with the team until consensus was reached.

## Results

The results of data extraction are reported in line with Aim I, Aim II, and Aim III.

### Aim I: composition of literature

Table 2 provides a list of the 30 included publications and a summary of their composition. One publication was a dissertation chapter (37), while all other publications were peer-reviewed journal articles (*n* = 29). The earliest year of publication was 2013 (38–40), while the most recent was 2025 (36, 41). Sixty percent (*n* = 18) of the publications were published within the last 5 years prior to the database search (6, 16, 18, 36, 37, 41–53). Publications were from authors in 15 different countries; these were the United States (*n* = 7) (16, 18, 37, 40, 43, 54, 55), Canada (*n* = 4) (38, 56–58), China, (*n* = 3) (6, 45, 48), Sweden (*n* = 3) (51, 53, 59), Finland (*n* = 2) (39, 46), Germany (*n* = 2) (17, 50), Australia (*n* = 1) (36), Czech Republic (*n* = 1) (41), Denmark (*n* = 1) (60), Ireland (*n* = 1) (49), Italy (*n* = 1) (52), Japan (*n* = 1) (47), South Korea (*n* = 1) (44), Spain (*n* = 1) (42), and The Netherlands (*n* = 1) (61). Thirteen publications explicitly stated their study design (16–18, 36–38, 44, 46, 49, 51, 53, 59, 61), 14 did not state their study design (6, 40–43, 45, 47, 48, 50, 52, 56–58, 60), and three publications, which consisted of sub-studies, described the design of their parent studies (39, 54, 55). Publications often utilized multiple study designs for the same study. The utilized study designs were longitudinal (*n* = 13) (18, 36, 37, 43, 46, 47, 49, 52, 53, 55, 58–60), cohort (*n* = 12) (38, 43, 47, 49–52, 54, 56, 58–60), cross-sectional (*n* = 10) (6, 17, 40–42, 44, 50, 56, 57, 59), observational (*n* = 9) (36, 38, 41–43, 47, 49, 58, 61), case-control (*n* = 4) (16, 45, 46, 48), nested (*n* = 2) (16, 55), randomized controlled trial (*n* = 2) (39, 46), prospective (*n* = 1) (49), and non-randomized experimental (*n* = 1) (18). Total sample sizes ranged widely [range = 10–746, mean (*M*) = 144, median (*Mdn*) = 85].

**Table 2:** Included publications and composition of literature.

Reference	Title	Publication type	University or Organization	Ethics approvals	Country	Journal	Study design	Total human sample size (*N*)
Arenas-Montes et al. (42)	Owning a pet is associated with changes in the composition of gut microbiota and could influence the risk of metabolic disorders in humans	Peer-reviewed	Reina Sofia University Hospital; University of Cordoba; Maimonides Biomedical Research Institute of Cordoba; Instituto de Salud Carlos III	Secondary data; not stated	Spain	Animals	Cross sectional study; observational study	162
Arishi et al. (36)	Development of the breastfed infant oral microbiome over the first 2 years of life in the BLOSOM Cohort	Peer-reviewed	The University of Western Australia; Australian Breastfeeding + Lactation Research and Science Translation (ABREAST) Network; The University of Western Australia Center for Human Lactation Research and Translation	Human Research Ethics Committee (Australia)	Australia	Frontiers in Cellular and Infection Microbiology	Longitudinal study; Observational study	84
Azad et al. (38)	Infant gut microbiota and the hygiene hypothesis of allergic disease: Impact of household pets and siblings on microbiota composition and diversity	Peer-reviewed	University of Alberta; Manitoba Institute of Child Health	Secondary data; University of Manitoba Human Research Ethics Board (Canada)	Canada	Allergy, Asthma and Clinical Immunology	Cohort study; Observational study	24
Birzele et al. (17)	Environmental and mucosal microbiota and their role in childhood asthma	Peer-reviewed	Ludwig Maximilian University of Munich	Secondary data	Germany	Allergy	Cross sectional study	86
Boolchandani et al. (37)	Understand and predict microbiome and resistome dynamics in response to perturbations across diverse populations and environments	Dissertation	Washington University in St. Louis	IRB; IACUC	United States	Arts and Sciences Electronic Theses and Dissertations	Longitudinal study	14
Dalton et al. (43)	Microbial sharing between pediatric patients and therapy dogs during hospital animal-assisted intervention programs	Peer-reviewed	Johns Hopkins University Bloomberg School of Public Health	IRB; IACUC; Scientific review committee	United States	Microorganisms	Cohort study; Longitudinal study; Observational study	49
Do et al. (44)	Comparative analysis of gut microbiota in humans living with and without companion animals	Peer-reviewed	Chungbuk National University; GutBiomeTech Co., LTD	IRB	South Korea	Life	Cross sectional study	120
Du et al. (45)	Effects of cat ownership on the gut microbiota of owners	Peer-reviewed	Hainan Medical University	Secondary data; Not stated	China	PLoS ONE	Case-control study	428
Gómez-Gallego et al. (46)	The composition and diversity of the gut microbiota in children is modifiable by the household dogs: Impact of a canine-specific probiotic	Peer-reviewed	University of Eastern Finland; Vetcare Ltd.	Ethics Committee (Finland)	Finland	Microorganisms	Case-control study; Longitudinal study	55
Ito et al. (47)	Comparative analysis based on shared amplicon sequence variants reveals that cohabitation influences gut microbiota sharing between humans and dogs	Peer-reviewed	Azabu University	Animal Ethics Committee (Japan); Ethical Committee for Medical and Health Research Involving Human Subjects (Japan)	Japan	Frontiers in Veterinary Science	Cohort study; Longitudinal study; Observational study	28
Jiang et al. (48)	Effects of dog ownership on the gut microbiota of elderly owners	Peer-reviewed	Hainan Medical University	Secondary data; Not stated	China	PLoS ONE	Case-control study	108
Kates et al. (16)	Household pet ownership and the microbial diversity of the human gut microbiota	Peer-reviewed	University of Wisconsin-Madison; William S. Middleton Veterans Hospital Madison	IRB; Secondary data	United States	Frontiers in Cellular and Infection Microbiology	Case-control study; Nested study	332
Kennedy et al. (59)	Oral microbiota development in early childhood	Peer-reviewed	Uppsala University	Secondary data; Regional Ethical Review Board (Sweden)	Sweden	Scientific Reports	Cohort study; Cross sectional study; Longitudinal study	118
Konya et al. (56)	Associations between bacterial communities of house dust and infant gut	Peer-reviewed	University of Toronto	Human Research Ethics Board (Canada)	Canada	Environmental Research	Cohort study; Cross sectional study	20
Korpela et al. (49)	Association between gut microbiota development and allergy in infants born during pandemic-related social distancing restrictions	Peer-reviewed	University College Cork	National COVID-19 Ethics Committee (Ireland)	Ireland	Allergy	Cohort study; Longitudinal study; Observational study; Prospective study	694
Langgartner et al. (50)	Association of the salivary microbiome with animal contact during early life and stress-induced immune activation in healthy participants	Peer-reviewed	University of Ulm	Ethics Committee (Germany)	Germany	Frontiers in Psychiatry	Cohort study; Cross sectional study	40
Laursen et al. (60)	Having older siblings is associated with gut microbiota development during early childhood	Peer-reviewed	National Food Institute; Technical University of Denmark	Secondary data; Committees on Biomedical Research Ethics (Denmark)	Denmark	BMC Microbiology	Cohort study; Longitudinal study	107
Levin et al. (54)	Joint effects of pregnancy, sociocultural, and environmental factors on early life gut microbiome structure and diversity	Peer-reviewed	Henry Ford Health System	IRB; Secondary data	United States	Scientific Reports	Cohort study	298
Ljung et al. (51)	Gut microbiota markers in early childhood are linked to farm living, pets in household and allergy	Peer-reviewed	University of Gothernburg; Statistiska Konsultgruppen; Skaraborg Hospital; Vara Clinical Center	Human Research Ethics Committee (Sweden)	Sweden	PLoS ONE	Cohort study	65
Martin et al. (61)	Early-life events, including mode of delivery and type of feeding, siblings and gender, shape the developing gut microbiota	Peer-reviewed	Nutricia Research	IRB	The Netherlands	PLoS ONE	Observational study	108
Misic et al. (55)	The shared microbiota of humans and companion animals as evaluated from *Staphylococcus* carriage sites	Peer-reviewed	University of Pennsylvania	IRB; IACUC	United States	Microbiome	Longitudinal study; Nested study	30
Nermes et al. (39)	Perinatal pet exposure, fecal microbiota, and wheezy bronchitis: is there a connection?	Peer-reviewed	Turku University Hospital	Not stated	Finland	International Scholarly Research Notices Allergy	Randomized controlled trial	27
Panzer et al. (18)	The impact of prenatal dog keeping on infant gut microbiota development	Peer-reviewed	Henry Ford Health System	IRB	United States	Clinical and Experimental Allergy	Non-randomized experimental study; Longitudinal study	131
Pavlíčková et al. (41)	Shaping the human gut microbiota: The role of canine companionship, lifestyle choices, and *Blastocystis* sp.	Peer-reviewed	Czech Academy of Sciences	Secondary data; World Medical Association's Declaration of Helsinki and Ethics Committee of the Biology Center of the Czech Academy of Sciences (Czech Republic)	Czech Republic	One Health	Cross sectional study; Observational study	118
Ross et al. (57)	The skin microbiome of cohabiting couples	Peer-reviewed	University of Waterloo	Office of Research Ethics (Canada)	Canada	mSystems	Cross sectional study	20
Scicchitano et al. (52)	A 15-day pilot biodiversity intervention with horses in a farm system leads to gut microbiome rewilding in 10 urban Italian children	Peer-reviewed	University of Bologna	Bioethics Committee (Bologna)	Italy	One Health	Cohort study; Longitudinal study	10
Song et al. (40)	Cohabiting family members share microbiota with one another and with their dogs	Peer-reviewed	University of Colorado, Boulder	IRB; IACUC	United States	eLife	Cross sectional study	159
Tun et al. (58)	Exposure to household furry pets influences the gut microbiota of infants at 3–4 months following various birth scenarios	Peer-reviewed	University of Alberta	University of Alberta Ethics Board (Canada)	Canada	Microbiome	Cohort study; Longitudinal study; Observational study	746
Wernroth et al. (53)	Development of gut microbiota during the first 2 years of life	Peer-reviewed	Uppsala University	Secondary data; Regional Ethics Review Board (Sweden)	Sweden	Scientific Reports	Longitudinal study	83
Zhang et al. (6)	Impact of environmental characteristics on children's gut microbiota - A pilot study in assessing the role of indoor microbiome and metabolites	Peer-reviewed	South China Agricultural University; Fudan University; Shanghai Key Laboratory of Meteorology and Health	Secondary data; Medical Ethics Committee (China)	China	Environmental Research	Cross sectional study	56

IRB, Institutional Review Board; IACUC, Institutional Animal Care and Use Committee.

### Aim II: HAI characteristics and microbiome characteristics

Table 3 displays the demographics for the human participants in the included publications and Table 4 displays the demographics for the animal participants. Table 5 displays the characteristics of the studied HAIs in the included publications. Table 6 displays the characteristics of the studied microbiome in the included publications.

**Table 3:** Human demographics.

Reference	Age range [mean (*M*), standard deviation (*SD*), median (*Mdn*)]	Gender	Race	Ethnicity
Arenas-Montes et al. (42)	*M* = 63.32 years, *SD* = 8.45 years	HAI group, men (*n* = 72), women (*n* = 11); Control group, men (*n* = 11), women (*n* = 18)	Not stated	Not stated
Arishi et al. (36)	0–2 years	Female (*n* = 44, 52.3%)	Maternal ethnicity, Caucasian (*n* = 74, 88.1%), Other (*n* = 10, 11.9%)	Not stated
Azad et al. (38)	*M* = 17.4 weeks, *SD* = 3.2 weeks, range 3–4 months	Male (*n* = 12, 50%); Female (*n* = 12, 50%)	Not stated	Not stated
Birzele et al. (17)	<8 years old (23.6%); 8 to <9 years old (23%); 9 to <10 years old (27.3%); ≥10 years old (26.1%)	Female (49.5%), Male (50.5%)	Not stated	Not stated
Boolchandani et al. (37)	Not stated	Male (100%)	Not stated	Not stated
Dalton et al. (43)	*M* = 11.68 years, *SD* = 4.7 years, range 1.9-20.4 years	Male (*n* =31, 63%); Female (*n* = 18, 37%)	Not stated	Not stated
Do et al. (44)	HAI adult group (*M* = 41.10, *SD* = 5.60 years); HAI children group (*M* = 9.55, *SD* = 2.91 years); Control adult group (*M* = 42.63, *SD* = 3.99 years); Control children group (*M* = 9.90, *SD* = 2.05 years)	HAI adult group, male (*n* = 20), female (*n* = 20); HAI children group, male (*n* = 11), female (*n* = 9); Control adults group, male (*n* =20), female (*n* = 20); Control children group, male (*n* = 9), female (*n* = 11)	Not stated	Not stated
Du et al. (45)	HAI group (*M* = 45.1 years, *SD* = 7.5 years); Control group (*M* = 46.1 years, *SD* = 15.4 years)	HAI group, female (*n* = 111), male (*n* = 103); Control group, female (*n* = 111) female, male (*n* = 103)	Caucasian (100%)	United Kingdom, United States
Gómez-Gallego et al. (46)	*M* = 2.28 years, range 1 month-5.33 years	Not stated	Not stated	Not stated
Ito et al. (47)	20-72 years (*M* = 48.5, *SD* = 15.7)	Males (*n* =13); Females (*n* = 15)	Not stated	Not stated
Jiang et al. (48)	HAI group (*M* = 69.78, *SD* = 3.15); Control group (*M* = 69.72, *SD* = 3.18)	HAI group, female (*n* = 14), male (*n* = 30); Control group, female (*n* = 14), male (*n* = 30)	Not stated	Not stated
Kates et al. (16)	Total (Mdn = 58 years, range 18-94 years); HAI group (Mdn = 53 years, range 18-93 years); Control group (Mdn = 63 years, range 19-94 years)	HAI group, male (*n* = 70, 39.3%), female (*n* = 108, 60.7%); Control group, male (*n* = 63, 40.9%), female (*n* = 91, 59.1%)	HAI group, Caucasian (*n* = 158, 88.8%), African American (*n* = 8, 4.5%), Other (*n* = 12, 6.7%); Control group, Caucasian (*n* = 127, 82.5%), African American (*n* = 19, 12.3%), Other (*n* = 8, 5.2%)	Not stated
Kennedy et al. (59)	0-2 years	Female (*n* = 22, 37%); Male (*n* = 37, 63%)	Not stated	Not stated
Konya et al. (56)	3-4 months	Not stated	Not stated	Not stated
Korpela et al. (49)	0-24 months	Male (55%)	Caucasian (96%)	Irish
Langgartner et al. (50)	20-40 years	Male (100%)	Not stated	Not stated
Laursen et al. (60)	9-18 months	Not stated	Not stated	Danish
Levin et al. (54)	1 month (*n* = 130, range 0.5-4.5); 6 months (*n* = 168, range 5.6-10.6)	Not stated	Caucasian (n=67, 21.8%); African American (n=191, 62.0%); Hispanic (n=22, 7.1%); Arab American (n=17, 5.5%); Other (n=11, 3.6%)	Not stated
Ljung et al. (51)	0-18 months	Girls (48%); Boys (52%)	Not stated	Not stated
Martin et al. (61)	First defecation; 2 days after first defecation; 1 week after birth; 1 month after birth; 3 months after birth; 6 months after birth; 1 week after weaning started	Boys (*n* = 56, 51.9%); Girls (*n* = 52, 48.1%)	Not stated	Not stated
Misic et al. (55)	HAI index group (Mdn = 13, range 6-15 years); HAI non-index group (Mdn = 26, range 3-54 years); Control index group (Mdn = 5, range 2-48 years); Control non-index group (Mdn = 23, range 9-42 years)	HAI group, male (*n* = 11), female (*n* = 9); Control group, male (*n* = 4), female (*n* = 6)	Not stated	Not stated
Nermes et al. (39)	1 month	Male (*n* = 61, 47.3%); Female (*n* = 68, 52.7%)	Not stated	Not stated
Panzer et al. (18)	0-18 months	Male (*n* = 68, 48.6%); Female (*n* = 72, 51.4%)	African American (*n* = 29, 20.6%)	Not stated
Pavlíčková et al. (41)	Not stated	Not stated	Not stated	Czech
Ross et al. (57)	20-49 years old	Male (50%); Female (50%)	Caucasian (*n* = 18); East Asian (*n* = 1), Asian (*n* = 1)	Not stated
Scicchitano et al. (52)	9-14 years	Not stated	Not stated	Italian
Song et al. (40)	HAI adults (range 18-59 years); HAI infants (range 0-12 months); HAI adolescents (range 1-17 years); HAI seniors (range 60+ years); Control adults (range 18-59); Control infants (range 0-12 months); Control adolescents (range 1-17 years); Control seniors (range 60+ years)	HAI adult group, female (*n* = 42), male (*n* = 42); HAI infant group, male (*n* = 3), female (*n* = 6); HAI adolescent group, male (*n* = 11), female (*n* = 6); HAI senior group, male (*n* = 4), female (*n* = 3)l Control adult group, male (*n* = 11), female (*n* = 11), Control infant group, male (*n* = 0), female (*n* = 3); Control adolescent group, male (*n* = 4), female (*n* = 5); Control senior group, male (*n* = 4), female (*n* = 4)	Not stated	Not stated
Tun et al. (58)	*M* = 3.34 months, range 2.7-4.3	Not stated	Maternal race, Caucasian (*n* = 561, 75.3%); Asian (*n* = 106, 14.2%); Other (*n* = 78, 10.5%)	Not stated
Wernroth et al. (53)	0-2 years	Boys (*n* = 50, 60%); Girls (*n* = 33, 40%)	Not stated	Not stated
Zhang et al. (6)	3-11 years	Female (*n* = 35, 62%); Male (*n* = 21, 38%)	Not stated	Not stated

**Table 4:** Animal demographics.

Reference	Age Range (mean *[M]*, standard deviation *[SD]*, median [*Mdn*])	Species	Breed	Relation to human
Arenas-Montes et al. (42)	Not stated	Dogs, cats, birds, other (including foxes, turtles, fishes, etc.)	Not stated	Companion animal
Arishi et al. (36)	Not stated	Not stated	Not stated	Companion animal
Azad et al. (38)	Not stated	Dogs, cats	Not stated	Companion animal
Birzele et al. (17)	Not stated	Farm animals, including cows	Not stated	Farm animal on farm where human lives
Boolchandani et al. (37)	Not stated	Swine	Not stated	Farm animal on farm where human interns
Dalton et al. (43)	*M* = 6.43 years, range 1.5-12 years	Dogs	Not stated	Therapy animal
Do et al. (44)	Not stated	Dogs, cats	Not stated	Companion animal
Du et al. (45)	Not stated	Cats	Not stated	Companion animal
Gómez-Gallego et al. (46)	*M* = 6.1 years	Dogs	Chihuahua; Dachshund; Danish Swedish Farmdog; English Bulldog; Golden Retriever; Labrador Retriever; Lagotto; Short-Haired Collie; American Staffordshire Terrier; German Shephard; Landseer; Novascotian Retriever; Samoyed	Companion animal
Ito et al. (47)	Range 1-10 years, *M* = 4.4, *SD* = 2.6	Dogs	Pure breed dog (*n* = 5); Mixed breed dog (*n* = 23)	Companion animal
Jiang et al. (48)	Not stated	Dogs	Not stated	Companion animal
Kates et al. (16)	Not stated	Dogs, cats, other pets (birds, rodents, reptiles)	Not stated	Companion animal
Kennedy et al. (59)	Not stated	Cats, dogs	Not stated	Companion animal
Konya et al. (56)	Not stated	Dogs, cats	Not stated	Companion animal
Korpela et al. (49)	Not stated	Not stated	Not stated	Companion animal
Langgartner et al. (50)	Not stated	Not stated	Not stated	Companion animal or farm animal on farm where human lives
Laursen et al. (60)	Not stated	Cats, dogs, rabbits	Not stated	Companion animal
Levin et al. (54)	Not stated	Cats, dogs	Not stated	Companion animal
Ljung et al. (51)	Not stated	Cats, dogs	Not stated	Companion animal
Martin et al. (61)	Not stated	Not stated	Not stated	Companion animal
Misic et al. (55)	Dogs: *Mdn* = 24 months, range 6-120 months; Cats: *Mdn* = 12 months, range 8-20 months	Cats, dogs, hamsters, ferrets, rabbits, sugar gliders	Not stated	Companion animal
Nermes et al. (39)	Not stated	“Mainly cats and dogs”	Not stated	Companion animal
Panzer et al. (18)	Not stated	Dogs	Not stated	Companion animal
Pavlíčková et al. (41)	Not stated	Dogs	Not stated	Companion animal
Ross et al. (57)	Not stated	Cats, dogs, fish	Not stated	Companion animal
Scicchitano et al. (52)	Not stated	Horses	Not stated	Farm animal at an educational farm the students are visiting
Song et al. (40)	Not stated	Dogs, cats, guinea pigs, fish, tarantulas, reptiles, amphibians, chickens, rabbits	Jack Russell Terrier (*n* = 1); Australian Cattle mix (*n* = 1); 1 Labrador/Golden mix; 1 Springer Spaniel; 1 Border Collie; 2 Border Collie mix; 1 Kelpie; 1 Standard Poodle; 2 Boxers; 5 Labradors; 1 English Setter; 1 Australian Shepherd/Spaniel mix; 1 German Shepherd/Malamute mix; 2 Australian Shepherds; 1 Bernese Mountain; 1 Siberian Husky; 1 Greater Swiss Mountain; 12 unknown dogs	Companion animal
Tun et al. (58)	Not stated	Dogs, cats, “other furry pets”	Not stated	Companion animal
Wernroth et al. (53)	Not stated	Cat, dog	Not stated	Companion animal
Zhang et al. (6)	Not stated	Not stated	Not stated	Companion animal

**Table 5:** Characteristics of human-animal interactions.

Reference	Number of animals	Time spent with animal	HAI sample size (*n*)	Control sample size (*n*)	Setting of interaction	Comparison condition
Arenas-Montes et al. (42)	Dogs (*n* = 56), cats (*n* = 19), birds, (*n* = 35), other (including foxes, turtles, fishes, etc.) (*n* = 11)	Not stated	83	79	Home of human and animal	No pet
Arishi et al. (36)	Not stated	Not stated	48	Not stated	Home of human and animal	No pet
Azad et al. (38)	Not stated	Not stated	15	9	Home of human and animal	No pet
Birzele et al. (17)	Not stated	Not stated	36	50	Farm where human and animal live	1. No farm exposure 2. No contact with cow or straw
Boolchandani et al. (37)	Not stated	3 months	14	14	Large-scale swine farm	Before/during/after
Dalton et al. (43)	Not stated	13 visits (*M* = 14 min per visit, range 2-42 min per visit)	49	49	Pediatric oncology outpatient unit	Before/after
Do et al. (44)	Dogs (*n* = 14), cats (*n* = 7)	At least 3 years	60	60	Home of human and animal	No pet
Du et al. (45)	Not stated	Not stated	214	214	Home of human and animal	No pet
Gómez-Gallego et al. (46)	Not stated	Not stated	37	18	Home of human and animal	No pet
Ito et al. (47)	Dog (*n* = 28)	2 weeks, 1 month, and 3 months	28	28	Home of human and animal	Before/after
Jiang et al. (48)	Not stated	Not stated	54	54	Home of human and animal	No pet
Kates et al. (16)	Dogs (*n* = 119), cats (*n* = 79), other pets (birds, rodents, reptiles) (*n* = 16)	Not stated	178	154	Home of human and animal	No pet
Kennedy et al. (59)	Not stated	Not stated	8	63	Home of human and animal	No pet
Konya et al. (56)	Not stated	Not stated	13	7	Home of human and animal	No pet
Korpela et al. (49)	Not stated	Not stated	Not stated	Not stated	Home of human and animal	No pet
Langgartner et al. (50)	Not stated	15 years (0-15 years of age)	20	20	Home of human and animal or farm where human and animal live	No animal contact, occasional animal contact, daily animal contact
Laursen et al. (60)	Not stated	Not stated	21	86	Home of human and animal	No pet
Levin et al. (54)	Not stated	At least 1 h a day	184	114	Home of human and animal	No pet
Ljung et al. (51)	Not stated	Not stated	40	25	Home of human and animal	No pet
Martin et al. (61)	Not stated	Not stated	Not stated	90	Home of human and animal	No pet
Misic et al. (55)	Cats (*n* = 3), dogs (*n* = 5), hamsters (*n* = 4), ferrets (*n* = 1), rabbits (*n* = 1), sugar gliders (*n* = 1)	Not stated	20	10	Home of human and animal	No pet
Nermes et al. (39)	Not stated	Not stated	17	10	Home of human and animal	Wheezing & no pet
Panzer et al. (18)	Not stated	At least 12 h a day, for at least 6 months prior to pregnancy, with plans to keep the dog for the study's duration	75	56	Home of human and animal	No pet
Pavlíčková et al. (41)	Not stated	Not stated	78	40	Home of human and animal	No pet
Ross et al. (57)	Cats (*n* = 8), dogs (*n* = 3), fish (*n* = 1)	Not stated	12	8	Home of human and animal	No pet
Scicchitano et al. (52)	Not stated	At least 10 h a day for 15 days	10	10	Educational farm	Before/after
Song et al. (40)	Not stated	Not stated	117	42	Home of human and animal	No pet
Tun et al. (58)	Not stated	Not stated	409	337	Home of human and animal	No pet
Wernroth et al. (53)	Not stated	Not stated	12	46	Home of human and animal	No pet
Zhang et al. (6)	Not stated	Not stated	50	*62*	Home of human and animal	No pet or plants

**Table 6:** Characteristics of microbiomes.

Reference	Microbiome location	16S rRNA sequencing used?	Microbial analysis method	Hypervariable region	Diversity indexes used
Arenas-Montes et al. (42)	Fecal	Yes	OTU clustering	V3-V4	Chao1, Shannon. Simpson
Arishi et al. (36)	Oral	Yes	OTU clustering	Full-length	Bray-Curtis, Shannon
Azad et al. (38)	Fecal	Yes	OTU clustering	V5-V7	Chao1, Shannon
Birzele et al. (17)	Nasal	Yes	OTU clustering	V3-V5	Bray-Curtis, Shannon, UniFrac
Boolchandani et al. (37)	Fecal	Yes	OTU clustering; whole-metagenome shotgun sequencing	V3-V4	Bray-Curtis, Shannon, UniFrac
Dalton et al. (43)	Nasal	Yes	ASV; OTU clustering	V1-V3	Faith's Phylogenetic, Shannon, UniFrac
Do et al. (44)	Fecal	Yes	OTU clustering	V3-V4	Bray-Curtis, Chao1, Shannon
Du et al. (45)	Fecal	Yes	OTU clustering	Not stated	Shannon
Gómez-Gallego et al. (46)	Fecal	Yes	ASV	V3-V4	Shannon
Ito et al. (47)	Fecal	Yes	ASV	V3-V4	Shannon, UniFrac
Jiang et al. (48)	Fecal	Yes	OTU clustering	Not stated	Shannon
Kates et al. (16)	Fecal	Yes	OTU clustering	V4	Bray-Curtis, Inverse Simpson, Shannon
Kennedy et al. (59)	Oral	Yes	OTU clustering	V3-V4	Inverse Simpson, Bray-Curtis
Konya et al. (56)	Fecal	Yes	OTU clustering	V5-V7	Not stated
Korpela et al. (49)	Fecal	Yes	Not stated	V3	Not stated
Langgartner et al. (50)	Saliva	Yes	OTU clustering	V4	Faith's Phylogenetic, Shannon, UniFrac
Laursen et al. (60)	Fecal	Yes	Not stated	V3	Shannon
Levin et al. (54)	Fecal	Yes	OTU clustering	V4	Faith's Phylogenetic, UniFrac
Ljung et al. (51)	Fecal	No	Quantitative culture	Not stated	Not stated
Martin et al. (61)	Fecal	No	qPCR	Not stated	Not stated
Misic et al. (55)	Skin (nares, pooled axillae/groin)	Yes	OTU clustering	V4	Bray-Curtis, Jaccard, Shannon, UniFrac
Nermes et al. (39)	Fecal	Not stated	Not stated	Not stated	Not stated
Panzer et al. (18)	Fecal	Yes	OTU clustering	V4	Canberra, Faith's Phylogenetic, UniFrac
Pavlíčková et al. (41)	Fecal	Yes	k-mer-based approach	V4	Bray-Curtis, Shannon
Ross et al. (57)	Skin (upper eyelids, outer nostrils, inner nostrils, armpits, torso, back, navel, inner thighs, bottom of feet, palms of hands)	Yes	OTU clustering	V3-V4	Bray-Curtis, Shannon
Scicchitano et al. (52)	Fecal	Yes	ASV; Shotgun sequencing; metagenome assembled genomes (MAGs) broken down into SGBs	V3-V4	Bray-Curtis, Faith's Phylogenetic, Shannon, Simpson, UniFrac
Song et al. (40)	Fecal; oral (dorsal tongue); skin (forehead, right palm, left palm)	Yes	OTU clustering	V2	Faith's Phylogenetic, UniFrac
Tun et al. (58)	Fecal	Yes	OTU clustering	V4	Chao1, Shannon, Simpson, UniFrac
Wernroth et al. (53)	Fecal; saliva	Yes	OTU clustering	V3-V4	Bray–Curtis, Inverse Simpson
Zhang et al. (6)	Fecal	Yes	ASV; OTU clustering	Not stated	Chao1, Shannon

OTU, Operational Taxonomic Unit; ASV, Amplicon Sequence Variant; qPCR, quantitative Polymerase Chain Reaction; SGB, Species-Level Genome Bin.

#### Human demographics

Twenty-eight publications provided age demographics for human participants. Of these, 18 focused exclusively on infants or children (6, 17, 18, 36, 38, 39, 43, 46, 49, 51–54, 56, 58–61), seven focused exclusively on adults (16, 42, 45, 47, 48, 50, 57), and three included both adults and children (40, 44, 55). Twenty-three publications provided gender demographics for human participants. Of these, two publications included exclusively men (37, 50), while the other 21 included multiple genders (6, 16–18, 36, 38–40, 42–45, 47–49, 51, 53, 55, 57, 59, 61). Eight publications provided race demographics (16, 18, 36, 45, 49, 54, 57, 58) and five publications provided ethnicity demographics (41, 45, 49, 52, 60).

#### Animal demographics

Animal demographics were more sparsely described than human demographics. Twenty-five articles described the species involved in the HAIs. Of these, the most commonly examined species were dogs (*n* = 21) (16, 18, 38–44, 46–48, 51, 53–60) and cats (*n* = 16) (16, 38–40, 42, 44, 45, 51, 53–60). Four studies described the species using terms such as “including cows,” “mainly cats and dogs,” and “other furry pets,” indicating there were likely other included species that were not provided in the publication (17, 39, 42, 58). Three studies provided information on the breed of the included dogs (40, 46, 47). Four studies provided information on the age ranges of studied animals, specifically three with information on dogs (43, 46, 47) and one with information on both dogs and cats (55). The types of animals studied were companion animals (*n* = 25) (6, 16, 18, 36, 38–42, 44–49, 51, 53–61), farm animals (*n* = 3) (17, 37, 52), therapy animals (*n* = 1) (43), and one study that included both companion and farm animals (50).

#### Human-Animal interaction characteristics

Some HAI characteristics were also sparsely described, with only six publications describing the number of animals involved in the interaction (16, 42, 44, 47, 55, 57) and only eight publications describing how much time was spent interacting with the animals (18, 37, 43, 44, 47, 50, 52, 54). Nearly all publications (*n* = 27) gave sample sizes for both the HAI group (range = 8–409, *M* = 67) and the control group (range = 8–337, *M* = *63*) (6, 16–18, 37–48, 50–59, 61). However, one study combined participants exposed to animals and participants exposed to plants, so it is unclear how many participants were exposed to animals specifically (6). Another study included participants with occasional contact with animals in their control group (50). The types of HAIs studied were interacting with companion animals (*n* = 25) (6, 16, 18, 36, 38–42, 44–49, 51, 53–61), living on a farm (*n* = 1) (17), interning on a farm (*n* = 1) (37), visiting an educational farm for humane education programming (*n* = 1) (52), hospital therapy animal visits (*n* = 1) (43), and one study that included both living on a farm and interacting with companion animals (50).

#### Microbiome characteristics

The human microbiomes measured in the included publications were fecal (*n* = 23) (6, 16, 18, 37–42, 44–49, 51–54, 56, 58, 60, 61), oral (*n* = 3) (36, 40, 59), skin (*n* = 3) (40, 55, 57), nasal (*n* = 2) (17, 43), and saliva (*n* = 2) (50, 53). Nearly all publications measured only one sample microbiome type, but one study examined both fecal and saliva microbiomes (53), and one study examined fecal, oral, and skin microbiomes (40). Most studies utilized 16s rRNA sequencing (*n* = 27) (6, 16–18, 36–38, 40–50, 52–60), while two studies utilized qPCR (39, 61), and one study utilized quantitative culture (51). Of the studies utilizing 16s rRNA sequencing, eighteen used solely operational taxonomic units (OTUs) clustering (16–18, 36, 38, 40, 42, 44, 45, 48, 50, 53–59), two used solely Amplicon Sequence Variants (ASVs) (46, 47), two used both OTU clustering and ASVs (6, 43), one used both OTU clustering and whole-metagenome shotgun sequencing (37), one used a k-mer-based approach (41), and one used ASVs, whole-metagenome shotgun sequencing, and metagenome assembled genomes broken down into Species-Level Genome Bins (SGBs) (52). Two studies did not state their approach (49, 60). Most publications included the V3 and/or the V4 hypervariable regions in their analysis (*n* = 20) (16–18, 37, 41–44, 46, 47, 49, 50, 52–55, 57–60).

### Aim III: outcomes on microbiome due to human-animal interactions

Table 7 displays the impacts of HAIs on human microbiomes found in the studies. Twenty-four studies reported a statistically significant effect of HAI on microbiome composition (6, 18, 36–40, 43–46, 48–55, 57–61), though two noted that significance varied depending on the measure used (18, 43). Of these twenty-four studies, significant differences included either more diversity in the microbiomes of the HAI group (*n* = 9) (6, 18, 38, 40, 43, 52, 55, 57, 58), or differences in microbiome composition between groups with no difference in their diversity (*n* = 14) (36, 37, 39, 44–46, 48–50, 53, 54, 59–61). One of the statistically significant studies found significant differences in the timing and frequency of microbial colonization between groups (51). Four studies did not find statistically significant differences between groups (16, 17, 41, 47). Two studies noted differences in microbial composition between groups, but significance levels are unclear (42, 56).

**Table 7:** Outcomes on microbiome due to human-animal interactions.

Reference	Outcome	Were outcomes significant?
Arenas-Montes et al. (42)	Microbiomes between groups were different, but it is unclear if one is more diverse than the other or if they are equally diverse	Unclear; Authors do not state significance levels but do note differences
Arishi et al. (36)	Microbiomes between groups were different, but neither was considered more diverse	Yes
Azad et al. (38)	HAI microbiomes were more diverse	Yes
Birzele et al. (17)	Microbiomes between groups were different, but neither was considered more diverse	No
Boolchandani et al. (37)	Microbiomes between groups were different, but neither was considered more diverse	Yes
Dalton et al. (43)	HAI microbiomes were more diverse	Yes; Note per some measures there were no significant differences
Do et al. (44)	Microbiomes between groups were different, but neither was considered more diverse	Yes
Du et al. (45)	Microbiomes between groups were different, but neither was considered more diverse	Yes
Gómez-Gallego et al. (46)	Microbiomes between groups were different, but neither was considered more diverse	Yes
Ito et al. (47)	Microbiomes between groups were different, but neither was considered more diverse	No
Jiang et al. (48)	Microbiomes between groups were different, but neither was considered more diverse	Yes
Kates et al. (16)	Minimal difference in microbiomes between groups	No
Kennedy et al. (59)	Microbiomes between groups were different, but neither was considered more diverse	Yes
Konya et al. (56)	Microbiomes between groups were different, but neither was considered more diverse	Unclear; Authors do not state significance levels for stool samples but do note differences
Korpela et al. (49)	Microbiomes between groups were different, but neither was considered more diverse	Yes
Langgartner et al. (50)	Microbiomes between groups were different, but neither was considered more diverse	Yes
Laursen et al. (60)	Microbiomes between groups were different, but neither was considered more diverse	Yes
Levin et al. (54)	Microbiomes between groups were different, but neither was considered more diverse	Yes
Ljung et al. (51)	Bacteria colonized at different times and at different frequencies for HAI compared to no HAI	Yes
Martin et al. (61)	Microbiomes between groups were different, but neither was considered more diverse	Yes
Misic et al. (55)	HAI microbiomes were more diverse	Yes
Nermes et al. (39)	Microbiomes between groups were different, but neither was considered more diverse	Yes
Panzer et al. (18)	HAI microbiomes were more diverse	Yes; Note per some measures there were no significant differences
Pavlíčková et al. (41)	HAI microbiomes were more diverse	No
Ross et al. (57)	HAI microbiomes were more diverse	Yes
Scicchitano et al. (52)	HAI microbiomes were more diverse	Yes
Song et al. (40)	HAI microbiomes were more diverse	Yes
Tun et al. (58)	HAI microbiomes were more diverse	Yes
Wernroth et al. (53)	Microbiomes between groups were different, but neither was considered more diverse	Yes
Zhang et al. (6)	HAI microbiomes were more diverse	Yes

## Discussion

The overarching goal of this scoping review was to collate literature to understand the effects of intentional, non-occupational human-animal interactions on human microbiomes. Databases were searched from inception, which included over five decades of publications. However, over half of the publications included in this review were published within the last five years. Additionally, multiple empirical studies on this topic have been published after the date of the database search for this review (7, 62, 63), indicating increased interest in this topic. Recognizing this trend, Skoufos et al. (1) called for a holistic understanding of human microbiomes within the context of the One Health approach.

This scoping review contributes to this understanding and draws on current literature to provide implications and future directions. The objectives included describing the composition of the literature related to intentional human-animal interaction and the microbiome, summarizing the human-animal interactions being measured and the method of measuring the microbiome, and summarizing the reported outcomes related to human-animal interaction and the microbiome. Findings of this scoping review demonstrate the potential for HAIs to influence the human microbiome.

### Aim I: composition of literature

Aim I of this scoping review was to describe the composition of the literature related to intentional human-animal interaction and the microbiome. Findings indicated that interest in this topic has grown quickly in recent years, with over half of the reviewed articles published in the last five years. This rapid, recent growth is seen in literature on other topics in both HAI (64–66) and the microbiome (67, 68). Interest in this topic is also international, as evidenced by the authors of these articles representing fifteen different countries. Compared to many other recent HAI or microbiome reviews, this review represents articles from a much higher number of countries, even when other reviews look at more articles (24, 66, 69, 70). This rapid and diverse growth coincides with the increase in recognition of the importance of One Health across the globe and the bidirectional impacts of HAIs. The increase in studies specifically focused on the impact of HAIs on the microbiome reflects interest in One Health at the intersection of microbiology and macro-level interactions.

Study designs were highly varied, with the most commonly stated being longitudinal, cohort, and cross-sectional designs. In line with other HAI and microbiome studies, sample sizes were also highly varied, with a drastic difference between the smallest sample size, 10, and the largest sample size, 746 (66, 68–70). However, overall sample sizes per study tended to be <200, with ten studies having an overall sample size of <50. These smaller sample sizes are particularly notable, as both microbiome and HAI studies have been criticized for having small samples that may impact reliability (66, 70). It may be that these smaller sample sizes are reflective of the niche nature of many of the populations of interest, such as veterinary students at a farm (37) or pediatric oncology patients engaging with visitation dogs at a single facility (43). Indeed, the largest samples in this review are from articles that looked broadly at companion animals and that acted as nested or secondary studies that had access to large cohorts or data sets (16, 45, 49, 58). Some populations represent smaller proportions of the overall population, and are more challenging to recruit, thus obtaining large sample sizes may be impractical. However, alpha and beta diversity index results may be heavily influenced by sample size (71). Therefore, careful attention must be paid to which diversity index is used and how those results are reported.

### Aim II: HAI characteristics and microbiome characteristics

Aim II of this scoping review was to summarize the human-animal interactions being measured and the methods of measuring the microbiome. Findings for this aim demonstrated that key information on human demographics, animal demographics, and HAI characteristics were often not reported, or reported unclearly. Less than one third of the reviewed articles reported race or ethnicity. Considering that ethnicity has been tied to measurable variations in the microbiome, documenting the ethnicity of participants is important for a full understanding of microbiome composition changes (72). Documenting ethnicity seems even more salient considering the diversity of countries represented in this review.

While most articles reported human age data, the way that age was reported varied greatly. Some articles reported detailed statistics, some articles broke ages down by study group, and some reported only high-level information such as the age range of the full sample. Human demographics, such as age, impact both the composition of the microbiome, as well as its susceptibility to change (4, 73). Thus, this inconsistency in reporting demographics impacts not only a full understanding of an individual article's findings, but also hinders comparison across studies.

Animal demographics were reported even less often. The age range of the animals were provided in only four articles, and the breeds of animals were provided in only three articles. As with human microbiomes, age impacts composition of and susceptibility to change in animal microbiomes (74). With the variation in age reporting for the human samples and the lack of age reporting for animal samples, not only is it unclear how age impacts each group's microbiome composition separately, the complex interactions between human and animal microbiomes are made even more complicated to interpret. Additionally, while most articles reported species information, some articles used vague statements such as “mainly cats and dogs” or “other furry pets.” Details of the HAIs, such as number of animals and length of time spent with animals, were similarly sparsely reported. HAI studies have previously been criticized for high variability and unclear reporting, which make comparison between studies difficult (75). To ensure studies of HAI and microbiomes do not face similar concerns, efforts should be taken to thoroughly and accurately report human demographics, animal demographics, and HAI characteristics.

### Aim III: outcomes on microbiome due to human-animal interactions

Aim III of this scoping review was to summarize the reported outcomes related to human-animal interaction and the microbiome. The majority of included publications demonstrated a difference in human microbiomes, either between groups, or before and after interacting with animals. While the character of differences in microbiomes varied between publications (e.g., microbial diversity, microbiome community composition, timing and frequency), the consistent presence of changes is notable.

More specifically, the reported effects of HAI were heterogeneous in both nature and direction. Nine studies reported greater microbial diversity associated with HAI, whereas fourteen identified differences in microbial community composition without corresponding differences in diversity. One study identified differences in the timing and frequency of microbial colonization. Conversely, four studies reported no statistically significant differences, and two reported compositional differences for which statistical significance was unclear. Thus, the available literature more consistently supports an association between HAI and differences in microbiome composition than a uniform directional effect on microbial diversity. Importantly, the heterogeneity of study populations, HAI exposures, microbiome sites, sequencing and analytical approaches, and reported outcomes prevents meaningful comparison of effect magnitude across studies. Likewise, changes in microbial diversity or taxonomic composition should not, in themselves, be interpreted as clinically beneficial or detrimental without corresponding functional or health outcomes. The null findings are therefore also informative and underscore that microbiome responses to HAI are likely context-dependent rather than universal.

### Implications

It may be possible that HAIs, which are known to have direct biopsychosocial impacts on humans, are also producing indirect impacts via changes to the microbiome (25). Considering the known connection between the composition of the microbiome and human physical, mental, and behavioral health, it is imperative to understand this potential indirect pathway for the impact of HAIs (5, 76, 77). Evaluation of the microbiome can be incorporated into future studies of HAIs to create a fuller picture of their biopsychosocial impacts, and consideration of potential HAIs can be incorporated into future studies of human microbiomes to better understand their composition. Additionally, analysis of HAIs and the microbiome may aid clinicians, practitioners, and public health professionals in supporting their clients.

Well-designed evaluation of the microbiome may serve as a novel approach to developing a more robust understanding of the biopsychosocial health impacts of HAIs. However, there are some notable gaps in the current literature that should be addressed to develop a better understanding of the impacts of HAIs on the human microbiome. In general, publications were heavily focused on the microbial aspects of the analysis, while documentation of the human demographics, details of the HAIs, and information on the animals participating in the interactions was sporadic. This missing information hinders a full understanding of these findings.

### Limitations

Findings from this scoping review should be interpreted in the context of the following limitations. Given the diverse nature of HAIs, it was necessary to narrow the scope of this review to the impact of intentional, non-occupational HAIs on human microbiomes to ensure feasibility and cohesion. However, this resulted in exclusion of studies covering topics such as farm workers, veterinary staff, human-wildlife interactions, and impacts on animal microbiomes. In addition, the variety of interventions and study types necessitated that this review would be a scoping review in format. Consequently, there was no formal quantification of methodological rigor or risk of bias. Future studies would benefit from a thorough review of other types of HAIs, a review of animal microbiomes, and a systematic analysis of existing literature.

Additionally, the search strings resulted in a relatively large number of articles for manual review, which may have increased the risk of mis-categorization of articles. This was generally due to HAI terms also serving as acronyms in many fields, such as “pet” also serving as an acronym for “positron emission tomography” and “polyethylene terephthalate.” To minimize this risk, a research librarian was consulted for refinement of search strings in accordance with each database's search functions. Additionally, multiple team members reviewed each article, and disagreements were addressed as a group.

While the data extraction process was a collaborative effort between multiple researchers, the backwards and forwards citation checking was conducted by one team member. There is potential for this process to introduce bias in excluded or included articles. However, this team member was a key part of the data extraction process, and thus highly familiar with the already included articles and the inclusion/exclusion criteria. Additionally, the one article chosen for inclusion based on citation checking was approved by additional team members. Therefore, while there is potential for articles to be excluded erroneously, it is unlikely that the included article should have been excluded instead.

The lack of sufficient explanation of the methodology used in microbiome sequencing limits the comparability of these studies. While this scoping review lists 16S, OTU/ASV, WMS, qPCR, and culture approaches, other important details were lacking in most cases. Information on OTU vs. ASV pipelines, variable 16S regions, DNA extraction, sequencing depth, batch effects, statistical pipelines, and taxonomic databases can all change conclusions.

### Future directions

Future studies of the impact of HAIs on the microbiome should aim to involve an interdisciplinary team of collaborators that includes subject-matter experts (SMEs) on the microbiome, human-animal interactions, and human health. A thoughtfully composed team can design studies and report findings in a way that holistically integrates all three of these topics, ensuring generalizability and clinical usefulness. In particular, studies of biopsychosocial impacts of HAI should incorporate microbiome SMEs to ensure that they are getting a full picture of HAI impacts. Studies of human microbiomes should incorporate HAI SMEs to ensure clear reporting of animal and HAI information.

Additionally, future studies should consider expanding which HAIs and microbiomes are explored. Existing literature is focused primarily on human-companion animal interactions, with only minimal focus on other forms of intentional, non-occupational HAI. Studies should examine HAI types including service dogs, therapy animals, volunteer-based interactions, and educational interactions. Existing literature also primarily explored the fecal microbiome. This is likely a practical choice based on the ease and standardization of fecal microbiome sampling. However, this review included studies of non-fecal microbiomes with findings indicating significant impacts of HAI. This indicates that impacts on non-fecal human microbiomes are particularly under-explored and need to be further understood. Future microbiome studies should adhere to acceptable guidelines for reporting their results. The Strengthening the Organization and Reporting of Microbiome Studies (STORMS) reporting guidelines, or an equivalent, be included in Supplementary materials of future microbiome studies (78).

## Conclusion

This scoping review provides a timely contribution to understanding the impacts of HAIs on the human microbiome. This understanding can provide a more complete picture of human biopsychosocial wellbeing within a One Health perspective. Findings from this scoping review are relevant to both practitioners and researchers. These findings can contribute to research via supporting thorough, well-executed study designs. Additionally, these findings provide initial evidence that microbiome analysis may serve as a novel approach for practitioners to understand and support the wellbeing of their clients in a holistic way. Interest in this topic has grown significantly over the last five years and is expected to continue growing. In response, this scoping review illuminates the current literature on HAIs and the human microbiome, and lays a foundation for future research.
