# Supplementary material for: The microbiome and non-occupational human-animal interactions: a scoping review
Source: Front Public Health. 2026 Sep 8;14:1938888. doi: 10.3389/fpubh.2026.1938888 (PMC13595336; doi:10.3389/fpubh.2026.1938888)
Supplement: Supplementary file 1 [file Data_Sheet_1.docx]

**Aim 1: Composition of Literature**

**Article Demographics**

Title

First Author

Last Author

Year of Publication

Type of Publication

Relevant University/Organization

Country associated with corresponding author

Journal or Repository

**Study Characteristics**

Aim/Purpose/Objective/Research Question

Study design

Total Sample Size

Ethics Approvals

**Aim 2: Summarize HAI and microbiome measures**

**Animal Demographics**

Animal age range

Species of animal(s) in the interaction

Breed(s) of animal

Animals' living situation

Animal's relation to human

**Human Demographics**

Participant age range

Participant Gender

Participant Race

Participant Ethnicity

**Human-Animal Interactions Demographics**

Role of human in interaction

Role of animal in interaction

Setting of interaction

Time spent with animal

HAI Sample Size

Control Sample Size

Comparison Condition/Control

**Microbiome Characteristics**

Microbiome Location(s)

Biodiversity Index

Was 16S rRNA sequencing used?

Hypervariable Region

Microbial Analysis Method

**Aim 3: Summarize Reported Outcomes**

Were significant differences present between HAI and control group microbiomes?

What differences were seen between HAI and control group microbiomes?
