# Supplementary material for: The microbiome and non-occupational human-animal interactions: a scoping review
Source: Front Public Health. 2026 Sep 8;14:1938888. doi: 10.3389/fpubh.2026.1938888 (PMC13595336; doi:10.3389/fpubh.2026.1938888)
Supplement: Supplementary file 2 [file Data_Sheet_2.docx]

# Supplemental Materials

## Database Search Syntax - PubMed

**Microbiome Search Terms**

"Microbiota"[MeSH Terms] OR "Hygiene Hypothesis"[MeSH Terms] OR "Brain-Gut Axis"[MeSH Terms] OR "microbiome"[Title/Abstract] OR "Microbiota"[Title/Abstract] OR "hygiene theory"[Title/Abstract] OR "Hygiene Hypothesis"[Title/Abstract] OR "bacterial flora"[Title/Abstract] OR "gut flora"[Title/Abstract] OR "gut-brain"[Title/Abstract]

**Human-Animal Interaction Search Terms**

“alpaca therapy”[Title/Abstract:~0] OR "animal assisted"[Title/Abstract] OR "animal facilitated"[Title/Abstract] OR "animal intervention"[Title/Abstract] OR "animal therapy"[Title/Abstract] OR "assistance animal*"[Title/Abstract] OR "assistance dog*"[Title/Abstract] OR "assistance horse"[Title/Abstract:~0] OR "canine therapy"[Title/Abstract] OR "canine assisted"[Title/Abstract] OR "canine facilitated"[Title/Abstract] OR “care farm*”[Title/Abstract] OR “cat therapy”[Title/Abstract] OR "companion animal*"[Title/Abstract] OR "dog therapy"[Title/Abstract] OR "dog assisted"[Title/Abstract] OR "dog facilitated"[Title/Abstract] OR "dolphin therapy"[Title/Abstract:~0] OR "dolphin assisted"[Title/Abstract] OR "dolphin facilitated"[Title/Abstract:~0] OR “education* farm”[Title/Abstract] OR "equine assisted"[Title/Abstract] OR "Equine-Assisted Therapy"[MeSH Terms] OR "equine facilitated"[Title/Abstract] OR "equine therapy"[Title/Abstract] OR “guinea pig therapy”[Title/Abstract:~0] OR "hippotherapy"[Title/Abstract] OR “horse therapy”[Title/Abstract] OR "horseback riding"[Title/Abstract] OR "human animal bond"[Title/Abstract] OR "human animal"[Title/Abstract] OR "human animal interaction"[Title/Abstract] OR “humane education”[Title/Abstract:~0] OR “llama therapy”[Title/Abstract:~0] OR “mini* pig therapy”[Title/Abstract] OR "Pets"[MeSH Terms] OR "pet"[Title/Abstract] OR "Pets"[Title/Abstract] OR "pet assisted"[Title/Abstract] OR "pet facilitated"[Title/Abstract] OR "pet therapy"[Title/Abstract] OR “pig therapy”[Title/Abstract:~0] OR “rabbit therapy”[Title/Abstract:~0] OR “rat therapy”[Title/Abstract] OR "Service Animals"[MeSH Terms] OR "service animal*"[Title/Abstract] OR "service dog*"[Title/Abstract] OR "service horse"[Title/Abstract:~0] OR "therapeutic animal*"[Title/Abstract] OR "therapeutic dog*"[Title/Abstract] OR "therapeutic horse*"[Title/Abstract] OR “therapeutic cat*”[Title/Abstract] OR “therapeutic rabbit*”[Title/Abstract] OR “therapeutic guinea pig*”[Title/Abstract] OR “therapeutic rat*”[Title/Abstract] OR “therapeutic bird*”[Title/Abstract] OR “therapeutic pig*”[Title/Abstract] OR “therapeutic mini* pig*”[Title/Abstract] OR “therapeutic llama*”[Title/Abstract] OR “therapeutic alpaca*”[Title/Abstract] OR "therapeutic horseback"[Title/Abstract] OR "therapeutic pet*"[Title/Abstract] OR "therapeutic riding"[Title/Abstract] OR "Therapy Animals"[MeSH Terms] OR "therapy animal*"[Title/Abstract] OR "therapy dog*"[Title/Abstract] OR "therapy horse*"[Title/Abstract] OR “therapy cat*”[Title/Abstract] OR “therapy rabbit*”[Title/Abstract] OR “therapy guinea pig*”[Title/Abstract] OR “therapy rat*”[Title/Abstract] OR “therapy bird*”[Title/Abstract] OR “therapy pig*”[Title/Abstract] OR “therapy mini* pig*”[Title/Abstract] OR “therapy llama*”[Title/Abstract] OR “therapy alpaca*”[Title/Abstract] OR "therapy pet*"[Title/Abstract] OR "therapy with animals"[Title/Abstract:~0] OR “zoo animal*”[Title/Abstract]

## Database Search Syntax – CINAHL

**Microbiome Search Terms**

(MH "Microbiota+") OR (MH "Brain-Gut Axis")

OR TI ( "microbiome*" OR "microbiota" OR "hygiene theory" OR "hygiene hypothesis" OR "bacterial flora" OR "gut flora" OR “gut microbiota” OR "gut-brain" OR “brain gut” ) OR AB ( "microbiome*" OR "microbiota" OR "hygiene theory" OR "hygiene hypothesis" OR "bacterial flora" OR "gut flora" OR “gut microbiota” OR "gut-brain" OR “brain gut” ) OR SU ( "microbiome*" OR "microbiota" OR "hygiene theory" OR "hygiene hypothesis" OR "bacterial flora" OR "gut flora" OR “gut microbiota” OR "gut-brain" OR “brain gut” )

**Human-Animal Interaction Search Terms**

(MH "Human-Animal Interaction")  OR (MH "Human-Pet Bonding") OR (MH "Pets") OR (MH "Service Animals") OR (MH "Therapy Animals") OR (MH "Pet Therapy+")

OR TI ( "animal assisted" OR "animal facilitated" OR "animal intervention" OR "animal therapy" OR "assistance animal*" OR "assistance dog*" OR "assistance horse" OR "canine therapy" OR "canine assisted" OR "canine facilitated" OR “care farm*” OR "companion animal*" OR "dog therapy" OR "dog assisted" OR "dog facilitated" OR "dolphin therapy" OR "dolphin assisted" OR "dolphin facilitated" OR “education* farm” OR "equine assisted" OR "Equine-Assisted Therapy" OR "equine facilitated" OR "equine therapy" OR "hippotherapy" OR "horseback riding" OR "human animal bond" OR "human animal" OR "human animal interaction" OR “humane education” OR "Pets" OR "pet" OR "Pets" OR "pet assisted" OR "pet facilitated" OR "pet therapy" OR "Service Animals" OR "service animal*" OR "service dog*" OR "service horse" OR "therapeutic animal*" OR "therapeutic dog*" OR "therapeutic horse*" OR "therapeutic horseback" OR "therapeutic pet*" OR "therapeutic riding" OR "Therapy Animals" OR "therapy animal*" OR "therapy dog*" OR "therapy horse*" OR "therapy pet*" OR "therapy with animals" OR “zoo animal*” OR “cat therapy” OR “rabbit therapy” OR “guinea pig therapy” OR “rat therapy” OR “bird therapy” OR “pig therapy” OR “mini* pig therapy” OR “llama therapy” OR “alpaca therapy” OR “therapy cat*” OR “therapy rabbit*” OR “therapy guinea pig*” OR “therapy rat*” OR “therapy bird*” OR “therapy pig*” OR “therapy mini* pig*” OR “therapy llama*” OR “therapy alpaca*” OR “therapeutic cat*” OR “therapeutic rabbit*” OR “therapeutic guinea pig*” OR “therapeutic rat*” OR “therapeutic bird*” OR “therapeutic pig*” OR “therapeutic mini* pig*” OR “therapeutic llama*” OR “therapeutic alpaca*”)

OR AB ( "animal assisted" OR "animal facilitated" OR "animal intervention" OR "animal therapy" OR "assistance animal*" OR "assistance dog*" OR "assistance horse" OR "canine therapy" OR "canine assisted" OR "canine facilitated" OR “care farm*” OR "companion animal*" OR "dog therapy" OR "dog assisted" OR "dog facilitated" OR "dolphin therapy" OR "dolphin assisted" OR "dolphin facilitated" OR “education* farm” OR "equine assisted" OR "Equine-Assisted Therapy" OR "equine facilitated" OR "equine therapy" OR "hippotherapy" OR "horseback riding" OR "human animal bond" OR "human animal" OR "human animal interaction" OR “humane education” OR "Pets" OR "pet" OR "Pets" OR "pet assisted" OR "pet facilitated" OR "pet therapy" OR "Service Animals" OR "service animal*" OR "service dog*" OR "service horse" OR "therapeutic animal*" OR "therapeutic dog*" OR "therapeutic horse*" OR "therapeutic horseback" OR "therapeutic pet*" OR "therapeutic riding" OR "Therapy Animals" OR "therapy animal*" OR "therapy dog*" OR "therapy horse*" OR "therapy pet*" OR "therapy with animals" OR “zoo animal*” OR “cat therapy” OR “rabbit therapy” OR “guinea pig therapy” OR “rat therapy” OR “bird therapy” OR “pig therapy” OR “mini* pig therapy” OR “llama therapy” OR “alpaca therapy” OR “therapy cat*” OR “therapy rabbit*” OR “therapy guinea pig*” OR “therapy rat*” OR “therapy bird*” OR “therapy pig*” OR “therapy mini* pig*” OR “therapy llama*” OR “therapy alpaca*” OR “therapeutic cat*” OR “therapeutic rabbit*” OR “therapeutic guinea pig*” OR “therapeutic rat*” OR “therapeutic bird*” OR “therapeutic pig*” OR “therapeutic mini* pig*” OR “therapeutic llama*” OR “therapeutic alpaca*”)

OR SU ( "animal assisted" OR "animal facilitated" OR "animal intervention" OR "animal therapy" OR "assistance animal*" OR "assistance dog*" OR "assistance horse" OR "canine therapy" OR "canine assisted" OR "canine facilitated" OR “care farm*” OR "companion animal*" OR "dog therapy" OR "dog assisted" OR "dog facilitated" OR "dolphin therapy" OR "dolphin assisted" OR "dolphin facilitated" OR “education* farm” OR "equine assisted" OR "Equine-Assisted Therapy" OR "equine facilitated" OR "equine therapy" OR "hippotherapy" OR "horseback riding" OR "human animal bond" OR "human animal" OR "human animal interaction" OR “humane education” OR "Pets" OR "pet" OR "Pets" OR "pet assisted" OR "pet facilitated" OR "pet therapy" OR "Service Animals" OR "service animal*" OR "service dog*" OR "service horse" OR "therapeutic animal*" OR "therapeutic dog*" OR "therapeutic horse*" OR "therapeutic horseback" OR "therapeutic pet*" OR "therapeutic riding" OR "Therapy Animals" OR "therapy animal*" OR "therapy dog*" OR "therapy horse*" OR "therapy pet*" OR "therapy with animals" OR “zoo animal*” OR “cat therapy” OR “rabbit therapy” OR “guinea pig therapy” OR “rat therapy” OR “bird therapy” OR “pig therapy” OR “mini* pig therapy” OR “llama therapy” OR “alpaca therapy” OR “therapy cat*” OR “therapy rabbit*” OR “therapy guinea pig*” OR “therapy rat*” OR “therapy bird*” OR “therapy pig*” OR “therapy mini* pig*” OR “therapy llama*” OR “therapy alpaca*” OR “therapeutic cat*” OR “therapeutic rabbit*” OR “therapeutic guinea pig*” OR “therapeutic rat*” OR “therapeutic bird*” OR “therapeutic pig*” OR “therapeutic mini* pig*” OR “therapeutic llama*” OR “therapeutic alpaca*”)

## Database Search Syntax – PsycINFO

**Microbiome Search Terms**

DE "Gut-Brain Axis" OR DE "Gastrointestinal Microbiota"

OR TI ( "microbiome*" OR "microbiota" OR "hygiene theory" OR "hygiene hypothesis" OR "bacterial flora" OR "gut flora" OR "gut brain" )

OR AB ( "microbiome*" OR "microbiota" OR "hygiene theory" OR "hygiene hypothesis" OR "bacterial flora" OR "gut flora" OR "gut brain" )

OR KW ( "microbiome*" OR "microbiota" OR "hygiene theory" OR "hygiene hypothesis" OR "bacterial flora" OR "gut flora" OR "gut brain" )

**Human-Animal Interaction Search Terms**

DE "Interspecies Interaction" OR DE “Animal Assisted Therapy” OR DE “Nature-Based Interventions” OR DE “Equine Assisted Therapy” OR DE “Wilderness Experience” OR DE “Therapeutic Camps” OR DE “Pets” OR DE “Service Animals” OR DE “Cats” OR DE “Dogs” OR DE "Service Animals"

OR TI ( "animal assisted" OR "animal facilitated" OR "animal intervention" OR "animal therapy" OR "assistance animal*" OR "assistance dog*" OR "assistance horse" OR "canine therapy" OR "canine assisted" OR "canine facilitated" OR “care farm*” OR "companion animal*" OR "dog therapy" OR "dog assisted" OR "dog facilitated" OR "dolphin therapy" OR "dolphin assisted" OR "dolphin facilitated" OR “education* farm” OR "equine assisted" OR "Equine-Assisted Therapy" OR "equine facilitated" OR "equine therapy" OR "hippotherapy" OR "horseback riding" OR "human animal bond" OR "human animal" OR "human animal interaction" OR “humane education” OR "Pets" OR "pet" OR "Pets" OR "pet assisted" OR "pet facilitated" OR "pet therapy" OR "Service Animals" OR "service animal*" OR "service dog*" OR "service horse" OR "therapeutic animal*" OR "therapeutic dog*" OR "therapeutic horse*" OR "therapeutic horseback" OR "therapeutic pet*" OR "therapeutic riding" OR "Therapy Animals" OR "therapy animal*" OR "therapy dog*" OR "therapy horse*" OR "therapy pet*" OR "therapy with animals" OR “zoo animal*” OR “cat therapy” OR “rabbit therapy” OR “guinea pig therapy” OR “rat therapy” OR “bird therapy” OR “pig therapy” OR “mini* pig therapy” OR “llama therapy” OR “alpaca therapy” OR “therapy cat*” OR “therapy rabbit*” OR “therapy guinea pig*” OR “therapy rat*” OR “therapy bird*” OR “therapy pig*” OR “therapy mini* pig*” OR “therapy llama*” OR “therapy alpaca*” OR “therapeutic cat*” OR “therapeutic rabbit*” OR “therapeutic guinea pig*” OR “therapeutic rat*” OR “therapeutic bird*” OR “therapeutic pig*” OR “therapeutic mini* pig*” OR “therapeutic llama*” OR “therapeutic alpaca*” )

OR AB ( "animal assisted" OR "animal facilitated" OR "animal intervention" OR "animal therapy" OR "assistance animal*" OR "assistance dog*" OR "assistance horse" OR "canine therapy" OR "canine assisted" OR "canine facilitated" OR “care farm*” OR "companion animal*" OR "dog therapy" OR "dog assisted" OR "dog facilitated" OR "dolphin therapy" OR "dolphin assisted" OR "dolphin facilitated" OR “education* farm” OR "equine assisted" OR "Equine-Assisted Therapy" OR "equine facilitated" OR "equine therapy" OR "hippotherapy" OR "horseback riding" OR "human animal bond" OR "human animal" OR "human animal interaction" OR “humane education” OR "Pets" OR "pet" OR "Pets" OR "pet assisted" OR "pet facilitated" OR "pet therapy" OR "Service Animals" OR "service animal*" OR "service dog*" OR "service horse" OR "therapeutic animal*" OR "therapeutic dog*" OR "therapeutic horse*" OR "therapeutic horseback" OR "therapeutic pet*" OR "therapeutic riding" OR "Therapy Animals" OR "therapy animal*" OR "therapy dog*" OR "therapy horse*" OR "therapy pet*" OR "therapy with animals" OR “zoo animal*” OR “cat therapy” OR “rabbit therapy” OR “guinea pig therapy” OR “rat therapy” OR “bird therapy” OR “pig therapy” OR “mini* pig therapy” OR “llama therapy” OR “alpaca therapy” OR “therapy cat*” OR “therapy rabbit*” OR “therapy guinea pig*” OR “therapy rat*” OR “therapy bird*” OR “therapy pig*” OR “therapy mini* pig*” OR “therapy llama*” OR “therapy alpaca*” OR “therapeutic cat*” OR “therapeutic rabbit*” OR “therapeutic guinea pig*” OR “therapeutic rat*” OR “therapeutic bird*” OR “therapeutic pig*” OR “therapeutic mini* pig*” OR “therapeutic llama*” OR “therapeutic alpaca*” )

OR KW ( "animal assisted" OR "animal facilitated" OR "animal intervention" OR "animal therapy" OR "assistance animal*" OR "assistance dog*" OR "assistance horse" OR "canine therapy" OR "canine assisted" OR "canine facilitated" OR “care farm*” OR "companion animal*" OR "dog therapy" OR "dog assisted" OR "dog facilitated" OR "dolphin therapy" OR "dolphin assisted" OR "dolphin facilitated" OR “education* farm” OR "equine assisted" OR "Equine-Assisted Therapy" OR "equine facilitated" OR "equine therapy" OR "hippotherapy" OR "horseback riding" OR "human animal bond" OR "human animal" OR "human animal interaction" OR “humane education” OR "Pets" OR "pet" OR "Pets" OR "pet assisted" OR "pet facilitated" OR "pet therapy" OR "Service Animals" OR "service animal*" OR "service dog*" OR "service horse" OR "therapeutic animal*" OR "therapeutic dog*" OR "therapeutic horse*" OR "therapeutic horseback" OR "therapeutic pet*" OR "therapeutic riding" OR "Therapy Animals" OR "therapy animal*" OR "therapy dog*" OR "therapy horse*" OR "therapy pet*" OR "therapy with animals" OR “zoo animal*” OR “cat therapy” OR “rabbit therapy” OR “guinea pig therapy” OR “rat therapy” OR “bird therapy” OR “pig therapy” OR “mini* pig therapy” OR “llama therapy” OR “alpaca therapy” OR “therapy cat*” OR “therapy rabbit*” OR “therapy guinea pig*” OR “therapy rat*” OR “therapy bird*” OR “therapy pig*” OR “therapy mini* pig*” OR “therapy llama*” OR “therapy alpaca*” OR “therapeutic cat*” OR “therapeutic rabbit*” OR “therapeutic guinea pig*” OR “therapeutic rat*” OR “therapeutic bird*” OR “therapeutic pig*” OR “therapeutic mini* pig*” OR “therapeutic llama*” OR “therapeutic alpaca*” )

## Database Search Syntax - CAB Abstracts

**Microbiome Search Terms**

TS=( microbiome* OR microbiota OR "hygiene theory" OR "hygiene hypothesis" OR "bacterial flora" OR "gut flora" OR "gut brain" OR "brain gut" )

**Human-Animal Interaction Search Terms**

TS=( "animal assisted" OR "animal facilitated" OR "animal intervention" OR "animal therapy" OR "assistance animal*" OR "assistance dog*" OR "assistance horse" OR "canine therapy" OR "canine assisted" OR "canine facilitated" OR “care farm*” OR "companion animal*" OR "dog therapy" OR "dog assisted" OR "dog facilitated" OR "dolphin therapy" OR "dolphin assisted" OR "dolphin facilitated" OR “education* farm” OR "equine assisted" OR "Equine-Assisted Therapy" OR "equine facilitated" OR "equine therapy" OR "hippotherapy" OR "horseback riding" OR "human animal bond" OR "human animal" OR "human animal interaction" OR “humane education” OR "Pets" OR "pet" OR "Pets" OR "pet assisted" OR "pet facilitated" OR "pet therapy" OR "Service Animals" OR "service animal*" OR "service dog*" OR "service horse" OR "therapeutic animal*" OR "therapeutic dog*" OR "therapeutic horse*" OR "therapeutic horseback" OR "therapeutic pet*" OR "therapeutic riding" OR "Therapy Animals" OR "therapy animal*" OR "therapy dog*" OR "therapy horse*" OR "therapy pet*" OR "therapy with animals" OR “zoo animal*” OR “cat therapy” OR “rabbit therapy” OR “guinea pig therapy” OR “rat therapy” OR “bird therapy” OR “pig therapy” OR “mini* pig therapy” OR “llama therapy” OR “alpaca therapy” OR “therapy cat*” OR “therapy rabbit*” OR “therapy guinea pig*” OR “therapy rat*” OR “therapy bird*” OR “therapy pig*” OR “therapy mini* pig*” OR “therapy llama*” OR “therapy alpaca*” OR “therapeutic cat*” OR “therapeutic rabbit*” OR “therapeutic guinea pig*” OR “therapeutic rat*” OR “therapeutic bird*” OR “therapeutic pig*” OR “therapeutic mini* pig*” OR “therapeutic llama*” OR “therapeutic alpaca*” )

## Database Search Syntax – Web of Science

**Microbiome Search Terms**

TI=( microbiome* OR microbiota OR "hygiene theory" OR "hygiene hypothesis" OR "bacterial flora" OR "gut flora" OR "gut brain" OR "brain gut" ) OR AB=( microbiome* OR microbiota OR "hygiene theory" OR "hygiene hypothesis" OR "bacterial flora" OR "gut flora" OR "gut brain" OR "brain gut" ) OR AK=( microbiome* OR microbiota OR "hygiene theory" OR "hygiene hypothesis" OR "bacterial flora" OR "gut flora" OR "gut brain" OR "brain gut" )

**Human-Animal Interaction Search Terms**

TI=( "animal assisted" OR "animal facilitated" OR "animal intervention" OR "animal therapy" OR "assistance animal*" OR "assistance dog*" OR "assistance horse" OR "canine therapy" OR "canine assisted" OR "canine facilitated" OR “care farm*” OR "companion animal*" OR "dog therapy" OR "dog assisted" OR "dog facilitated" OR "dolphin therapy" OR "dolphin assisted" OR "dolphin facilitated" OR “education* farm” OR "equine assisted" OR "Equine-Assisted Therapy" OR "equine facilitated" OR "equine therapy" OR "hippotherapy" OR "horseback riding" OR "human animal bond" OR "human animal" OR "human animal interaction" OR “humane education” OR "Pets" OR "pet" OR "Pets" OR "pet assisted" OR "pet facilitated" OR "pet therapy" OR "Service Animals" OR "service animal*" OR "service dog*" OR "service horse" OR "therapeutic animal*" OR "therapeutic dog*" OR "therapeutic horse*" OR "therapeutic horseback" OR "therapeutic pet*" OR "therapeutic riding" OR "Therapy Animals" OR "therapy animal*" OR "therapy dog*" OR "therapy horse*" OR "therapy pet*" OR "therapy with animals" OR “zoo animal*” OR “cat therapy” OR “rabbit therapy” OR “guinea pig therapy” OR “rat therapy” OR “bird therapy” OR “pig therapy” OR “mini* pig therapy” OR “llama therapy” OR “alpaca therapy” OR “therapy cat*” OR “therapy rabbit*” OR “therapy guinea pig*” OR “therapy rat*” OR “therapy bird*” OR “therapy pig*” OR “therapy mini* pig*” OR “therapy llama*” OR “therapy alpaca*” OR “therapeutic cat*” OR “therapeutic rabbit*” OR “therapeutic guinea pig*” OR “therapeutic rat*” OR “therapeutic bird*” OR “therapeutic pig*” OR “therapeutic mini* pig*” OR “therapeutic llama*” OR “therapeutic alpaca*” )

OR AB=( "animal assisted" OR "animal facilitated" OR "animal intervention" OR "animal therapy" OR "assistance animal*" OR "assistance dog*" OR "assistance horse" OR "canine therapy" OR "canine assisted" OR "canine facilitated" OR “care farm*” OR "companion animal*" OR "dog therapy" OR "dog assisted" OR "dog facilitated" OR "dolphin therapy" OR "dolphin assisted" OR "dolphin facilitated" OR “education* farm” OR "equine assisted" OR "Equine-Assisted Therapy" OR "equine facilitated" OR "equine therapy" OR "hippotherapy" OR "horseback riding" OR "human animal bond" OR "human animal" OR "human animal interaction" OR “humane education” OR "Pets" OR "pet" OR "Pets" OR "pet assisted" OR "pet facilitated" OR "pet therapy" OR "Service Animals" OR "service animal*" OR "service dog*" OR "service horse" OR "therapeutic animal*" OR "therapeutic dog*" OR "therapeutic horse*" OR "therapeutic horseback" OR "therapeutic pet*" OR "therapeutic riding" OR "Therapy Animals" OR "therapy animal*" OR "therapy dog*" OR "therapy horse*" OR "therapy pet*" OR "therapy with animals" OR “zoo animal*” OR “cat therapy” OR “rabbit therapy” OR “guinea pig therapy” OR “rat therapy” OR “bird therapy” OR “pig therapy” OR “mini* pig therapy” OR “llama therapy” OR “alpaca therapy” OR “therapy cat*” OR “therapy rabbit*” OR “therapy guinea pig*” OR “therapy rat*” OR “therapy bird*” OR “therapy pig*” OR “therapy mini* pig*” OR “therapy llama*” OR “therapy alpaca*” OR “therapeutic cat*” OR “therapeutic rabbit*” OR “therapeutic guinea pig*” OR “therapeutic rat*” OR “therapeutic bird*” OR “therapeutic pig*” OR “therapeutic mini* pig*” OR “therapeutic llama*” OR “therapeutic alpaca*” )

OR AK=( "animal assisted" OR "animal facilitated" OR "animal intervention" OR "animal therapy" OR "assistance animal*" OR "assistance dog*" OR "assistance horse" OR "canine therapy" OR "canine assisted" OR "canine facilitated" OR “care farm*” OR "companion animal*" OR "dog therapy" OR "dog assisted" OR "dog facilitated" OR "dolphin therapy" OR "dolphin assisted" OR "dolphin facilitated" OR “education* farm” OR "equine assisted" OR "Equine-Assisted Therapy" OR "equine facilitated" OR "equine therapy" OR "hippotherapy" OR "horseback riding" OR "human animal bond" OR "human animal" OR "human animal interaction" OR “humane education” OR "Pets" OR "pet" OR "Pets" OR "pet assisted" OR "pet facilitated" OR "pet therapy" OR "Service Animals" OR "service animal*" OR "service dog*" OR "service horse" OR "therapeutic animal*" OR "therapeutic dog*" OR "therapeutic horse*" OR "therapeutic horseback" OR "therapeutic pet*" OR "therapeutic riding" OR "Therapy Animals" OR "therapy animal*" OR "therapy dog*" OR "therapy horse*" OR "therapy pet*" OR "therapy with animals" OR “zoo animal*” OR “cat therapy” OR “rabbit therapy” OR “guinea pig therapy” OR “rat therapy” OR “bird therapy” OR “pig therapy” OR “mini* pig therapy” OR “llama therapy” OR “alpaca therapy” OR “therapy cat*” OR “therapy rabbit*” OR “therapy guinea pig*” OR “therapy rat*” OR “therapy bird*” OR “therapy pig*” OR “therapy mini* pig*” OR “therapy llama*” OR “therapy alpaca*” OR “therapeutic cat*” OR “therapeutic rabbit*” OR “therapeutic guinea pig*” OR “therapeutic rat*” OR “therapeutic bird*” OR “therapeutic pig*” OR “therapeutic mini* pig*” OR “therapeutic llama*” OR “therapeutic alpaca*” )

## Database Search Syntax – ProQuest Dissertations & Theses

**Microbiome Search Terms**

SUMMARY,SUBJECT,TITLE(microbiome* OR microbiota OR "hygiene theory" OR "hygiene hypothesis" OR "bacterial flora" OR "gut flora" OR "gut brain" OR "brain gut" )

**Human-Animal Interaction Search Terms**

SUMMARY,SUBJECT,TITLE( "animal assisted" OR "animal facilitated" OR "animal intervention" OR "animal therapy" OR "assistance animal*" OR "assistance dog*" OR "assistance horse" OR "canine therapy" OR "canine assisted" OR "canine facilitated" OR “care farm*” OR "companion animal*" OR "dog therapy" OR "dog assisted" OR "dog facilitated" OR "dolphin therapy" OR "dolphin assisted" OR "dolphin facilitated" OR “education* farm” OR "equine assisted" OR "Equine-Assisted Therapy" OR "equine facilitated" OR "equine therapy" OR "hippotherapy" OR "horseback riding" OR "human animal bond" OR "human animal" OR "human animal interaction" OR “humane education” OR "Pets" OR "pet" OR "Pets" OR "pet assisted" OR "pet facilitated" OR "pet therapy" OR "Service Animals" OR "service animal*" OR "service dog*" OR "service horse" OR "therapeutic animal*" OR "therapeutic dog*" OR "therapeutic horse*" OR "therapeutic horseback" OR "therapeutic pet*" OR "therapeutic riding" OR "Therapy Animals" OR "therapy animal*" OR "therapy dog*" OR "therapy horse*" OR "therapy pet*" OR "therapy with animals" OR “zoo animal*” OR “cat therapy” OR “rabbit therapy” OR “guinea pig therapy” OR “rat therapy” OR “bird therapy” OR “pig therapy” OR “mini* pig therapy” OR “llama therapy” OR “alpaca therapy” OR “therapy cat*” OR “therapy rabbit*” OR “therapy guinea pig*” OR “therapy rat*” OR “therapy bird*” OR “therapy pig*” OR “therapy mini* pig*” OR “therapy llama*” OR “therapy alpaca*” OR “therapeutic cat*” OR “therapeutic rabbit*” OR “therapeutic guinea pig*” OR “therapeutic rat*” OR “therapeutic bird*” OR “therapeutic pig*” OR “therapeutic mini* pig*” OR “therapeutic llama*” OR “therapeutic alpaca*” )
